# Assessment of longitudinal brain development using super‐resolution magnetic resonance imaging following fetal surgery for open spina bifida

**DOI:** 10.1002/uog.26244

**Published:** 2023-11-01

**Authors:** N. Mufti, J. Chappell, M. Aertsen, M. Ebner, L. Fidon, J. Deprest, A. L. David, A. Melbourne, GIFT‐Surg Imaging Working Group, David Atkinson, Foteini Emmanouella Bredaki, Luc De Catte, Phillippe De Vloo, Philippe Demaerel, Roland Devlieger, Trevor Gaunt, Giles S. Kendall, Sebastien Ourselin, Kelly Pegoretti Baruteau, Adalina Sacco, Magdalena Sokolska, Dominic Thompson, Tom Vercauteren

**Affiliations:** ^1^ Elizabeth Garrett Anderson Institute for Women's Health University College London London UK; ^2^ School of Biomedical Engineering and Imaging Sciences (BMEIS) King's College London London UK; ^3^ Department of Radiology University Hospitals Katholieke Universiteit (KU) Leuven Leuven Belgium; ^4^ Department of Obstetrics and Gynaecology University Hospitals Katholieke Universiteit (KU) Leuven Leuven Belgium; ^5^ National Institute for Health and Care Research University College London Hospitals Biomedical Research Centre London UK; ^6^ Medical Physics and Biomedical Engineering University College London London UK

**Keywords:** curvedness, fetal surgery, gyrification, magnetic resonance imaging, MRI, open spina bifida, super‐resolution reconstruction, surface area, volume

## Abstract

**Objectives:**

Prenatal surgery is offered for selected fetuses with open spina bifida (OSB) to improve long‐term outcome. We studied the effect of fetal OSB surgery on brain development using advanced magnetic resonance imaging (MRI) techniques to quantify the volume, surface area and shape of cerebral structures and to analyze surface curvature by means of parameters that correspond to gyrification.

**Methods:**

We compared MRI data from 29 fetuses with OSB before fetal surgery (mean gestational age (GA), 23 + 3 weeks) and at 1 and 6 weeks after surgery, with that of 36 GA‐matched control fetuses (GA range, 21 + 2 to 36 + 2 weeks). Automated super‐resolution reconstruction provided three‐dimensional isotropic volumetric brain images. Unmyelinated white matter, cerebellum and ventricles were segmented automatically and refined manually, after which volume, surface area and shape parameter (volume/surface area) were quantified. Mathematical markers (shape index (SI) and curvedness) were used to measure gyrification. Parameters were assessed according to lesion type (myelomeningocele *vs* myeloschisis (MS)), postoperative persistence of hindbrain herniation (HH) and the presence of supratentorial anomalies, namely partial agenesis of the corpus callosum (pACC) and heterotopia (HT).

**Results:**

Growth in ventricular volume per week and change in shape parameter per week were higher at 6 weeks after surgery in fetuses with OSB compared with controls (median, 2500.94 (interquartile range (IQR), 1689.70–3580.80) mm^3^/week *vs* 708.21 (IQR, 474.50–925.00) mm^3^/week; *P* < 0.001 and 0.075 (IQR, 0.047–0.112) mm/week *vs* 0.022 (IQR, 0.009–0.042) mm/week; *P* = 0.046, respectively). Ventricular volume growth increased 6 weeks after surgery in cases with pACC (*P* < 0.001) and those with persistent HH (*P* = 0.002). During that time period, the change in unmyelinated white‐matter shape parameter per week was decreased in OSB fetuses compared with controls (0.056 (IQR, 0.044–0.092) mm/week *vs* 0.159 (IQR, 0.100–0.247) mm/week; *P* = 0.002), particularly in cases with persistent HH (*P* = 0.011), MS (*P* = 0.015), HT (*P* = 0.022), HT with corpus callosum anomaly (*P* = 0.017) and persistent HH with corpus callosum anomaly (*P* = 0.007). At 6 weeks postoperatively, despite OSB fetuses having a lower rate of change in curvedness compared with controls (0.061 (IQR, 0.040–0.093) mm^–1^/week vs 0.094 (IQR, 0.070–0.146) mm^–1^/week; *P* < 0.001), reversing the trend seen at 1 week after surgery (0.144 (IQR, 0.099–0.236) mm^–1^/week *vs* 0.072 (IQR, 0.059–0.081) mm^–1^/week; *P* < 0.001), gyrification, as determined using SI, appeared to be increased in OSB fetuses overall compared with controls. This observation was more prominent in fetuses with pACC and those with severe ventriculomegaly (*P*‐value range, < 0.001 to 0.006).

**Conclusions:**

Following fetal OSB repair, volume, shape and curvedness of ventricles and unmyelinated white matter differed significantly compared with those of normal fetuses. Morphological brain changes after fetal surgery were not limited to effects on the circulation of cerebrospinal fluid. These observations may have implications for postnatal neurocognitive outcome. © 2023 The Authors. *Ultrasound in Obstetrics & Gynecology* published by John Wiley & Sons Ltd on behalf of International Society of Ultrasound in Obstetrics and Gynecology.

**CONTRIBUTION**

*What are the novel findings of this work?*

Using novel magnetic resonance imaging post‐processing technology, we demonstrated differences in cerebral volume and shape after surgery in fetuses with open spina bifida (OSB) compared with control fetuses without OSB. Surface curvature analysis indicated higher gyrification in OSB fetuses, particularly in cases with partial corpus callosum agenesis and those with severe ventriculomegaly.

*What are the clinical implications of this work?*

Our findings suggest the presence of differences in folding, volume and shape in most layers of the cerebral cortex in fetuses with OSB compared with controls. Additional morphological changes after fetal surgery in the supratentorial compartment, such as altered gyrification, may be more predictive of neurocognitive outcome than reversal of hindbrain herniation alone.

## INTRODUCTION

Open spina bifida (OSB) is one of the most common congenital malformations of the central nervous system (CNS)^1^, ^2^, ^3^, ^4^. It is characterized by failure of the neural tube to close, leading to leakage of cerebrospinal fluid (CSF) which, in turn, results in hydrostatic pressure loss, causing Chiari‐II malformation stigmata^5^, ^6^, ^7^, ^8^. This includes caudal displacement of hindbrain structures, which is believed to contribute to obstructive hydrocephalus^2^, ^5^, ^7^, ^8^. Because of mechanical stretching of the brain parenchyma, hydrocephalus can predispose to supratentorial anomalies, such as corpus callosum hypoplasia or partial dysgenesis^8^, ^9^, ^10^, ^11^, ^12^, ^13^, ^14^, ^15^, ^16^, ^17^, ^18^. It can also cause disruption in cortical organization, leading to heterotopia and altered gyrification^5^, ^8^, ^16^. In children with OSB who undergo postnatal closure, increased gyrification measured on magnetic resonance imaging (MRI) has tended to be associated negatively with intelligence quotient and fine motor outcome^2^, ^10^.

The Management of Myelomeningocele Study (MOMS) provided Level‐I evidence that fetal OSB closure reduces the need for ventriculoperitoneal shunt by 40% and the extent of hindbrain herniation by 30%, by 12 months of age^4^, ^14^, ^19^. The improved outcome may be related to reversal of Chiari‐II malformation and halting of the progression of hydrocephalus^2^, ^14^. Given this evidence, fetal surgery is now offered to suitable fetal candidates^20^, ^21^, ^22^, ^23^.

Fetal MRI is an essential part of the presurgical work‐up for appropriate candidate selection and plays an important role in guiding parental counseling and postnatal management^6^, ^20^. MRI complements prenatal sonography and provides superior contrast resolution^24^, ^25^. Despite technical developments in MRI, in terms of rapid‐pulse sequences and advances in coil design, it is still limited by fetal motion artifacts^15^. In this study we used novel post‐processing techniques, including super‐resolution reconstruction (SRR), which can reduce fetal motion effects^2^, ^8^, ^10^, ^14^, ^26^, ^27^, ^28^, ^29^, ^30^. We hypothesized that this may improve quantitative cerebral assessment to aid our understanding of the impact of fetal OSB closure on brain development, which could be useful in ascertaining its outcome. We studied fetuses before, and approximately 1 and 6 weeks after, fetal OSB surgery using an automated SRR technique to reconstruct the fetal brain, from which we quantified cerebral, cerebellar and ventricular volumes and surface areas, and evaluated longitudinal cortical gyrification using surface curvature markers^2^, ^28^.

## METHODS

### Ethics

This was a prospective multicenter case–control study of women undergoing fetal surgery for OSB at two NHS England specialist commissioned fetal surgery centers (FSCs) at University College London Hospital (UCLH) and Universitair Ziekenhuis (UZ) Leuven^31^. All MRI data were analyzed under the study entitled ‘Guided instrumentation for fetal therapy and surgery (GIFT‐Surg): fetal MRI to improve prenatal diagnosis and therapy for fetal abnormality’, which was approved by Hampstead Research Ethics Committee (15/LO/1488) and the Ethics Committee for Clinical Research at UZ Leuven (S60814). The women provided written informed consent to participate in fetal MRI research. All images from the referring units were transferred to the FSC at UCLH via the Image Exchange Portal (Sectra Ltd, Linköping, Sweden). All images were then transferred with UCLH Caldicott Guardian approval to collaborators for analysis at partner academic institutions, University College London and King's College London, via the secure GIFT‐Cloud platform^32^, which ensures anonymization through eXtensible Neuroimaging Archive Toolkit (XNAT) technology. XNAT is an open‐source imaging software that aids imaging‐based research by facilitating the management, importing, archiving, processing and secure distribution of imaging data^32^.

### Dataset and fetal imaging

**Figure 1:**
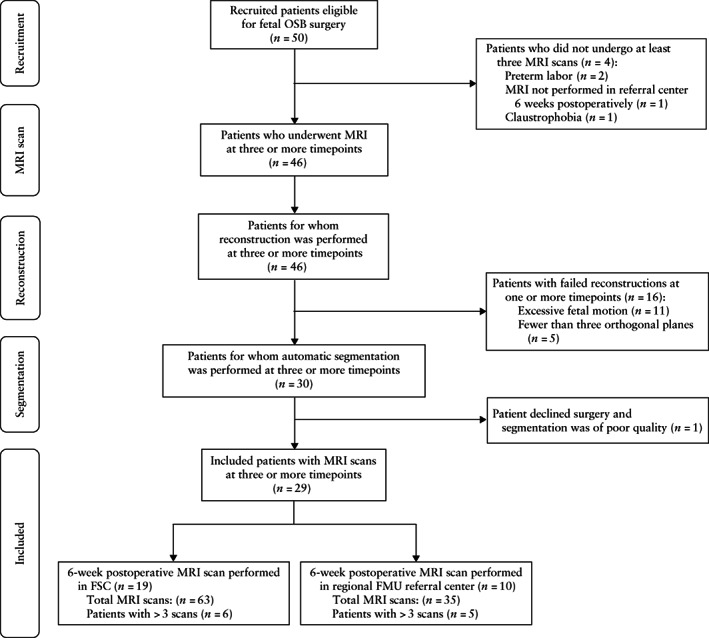
Flowchart summarizing recruitment of patients eligible for fetal open spina bifida (OSB) surgery, and acquisition, reconstruction and segmentation of magnetic resonance imaging (MRI) scans at three timepoints (approximately 1 week before, 1 week after and 6 weeks after surgery). If patient had more than three scans, highest‐quality reconstruction was used for each of three timepoints. FMU, fetal medicine unit; FSC, fetal surgery center.

Fifty women with fetal OSB were recruited for fetal surgery, in accordance with MOMS trial eligibility criteria^4^, ^22^. MRI evaluation was performed at three timepoints for each patient: before fetal surgery (usually before 24 weeks) and at approximately 1 and 6 weeks after surgery. The MRI scans at the first and second timepoints were performed in the FSC at UCLH or UZ Leuven. The MRI scan at the third timepoint was performed at either UCLH or the patient's regional fetal medicine unit (FMU) referral center, depending on their distance from, and ability to travel to, the FSC. Information about MRI acquisition and scan time was sourced from all imaging units. Four patients were excluded as they did not undergo a MRI scan 6 weeks postoperatively (two owing to preterm birth, one because MRI was not performed in the regional FMU referral center and the patient was unable to travel to the FSC and one owing to claustrophobia). For the remaining 46 patients, we reconstructed the fetal brain from the MRI scan at each timepoint. A further 16 patients were excluded owing to failed reconstructions with substantial artifact/blur at one or more timepoints. An additional patient was excluded because they declined surgery and segmentation was of poor quality despite successful reconstruction. Therefore, observations of 29 patients with scans at three or more timepoints were included (Figure 1). These were compared with 12 retrospectively recruited gestational‐age‐matched control fetuses that had no CNS abnormalities and were assessed for other congenital or placental anomalies (including lymphangioma and placenta accreta spectrum disorder), at each corresponding timepoint (a total of 36 control measurements). We assumed a linear trajectory of growth and a cross‐sectional representation of normal gyrification using our controls, based on previous postmortem histological evidence and *in‐vivo* MRI studies of fetal brain development^33^, ^34^, ^35^, ^36^. In the absence of data with higher temporal resolution, we considered that assuming a linear change in sulcation was appropriate.

MRI data were acquired on a 1.5‐Tesla system in FSCs at UCLH (MAGENTOM Avanto; Siemens Healthineers, Erlangen, Germany), UZ Leuven (MAGENTOM Aera; Siemens Healthineers) and in regional FMU referral centers, without maternal or fetal sedation and in the normal specific absorption rate operating mode. Two small body coils were placed adjacent to each other on the maternal abdomen, with the mother in the supine or left lateral decubitus position. In the FSCs, the protocol consisted of T2‐weighted fast acquisition spin‐echo sequences, typically half‐Fourier acquisition single‐shot turbo spin echo (HASTE), performed on the fetal brain in multiple orientations with at least one stack in each of the axial, coronal and sagittal planes. In the FMU referral centers, the protocol for fetal brain imaging consisted of either T2‐weighted HASTE, single‐shot fast‐spin echo or turbo‐spin echo in three orthogonal planes. MRI parameters for the above sequences are displayed in Table S1. Other sequences performed in all centers included echoplanar imaging, T1‐weighted imaging and diffusion‐weighted imaging. The total acquisition time for all scans was a maximum of 60 min.

### Automated super‐resolution reconstruction

**Figure 2:**
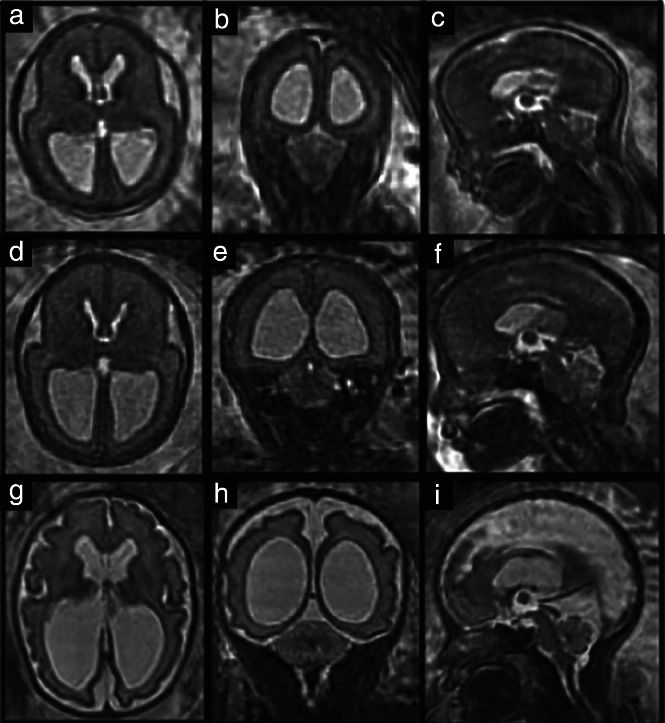
Original super‐resolution reconstruction data in fetus with open spina bifida, obtained before fetal surgery (23 + 3 weeks' gestation) (a–c), approximately 1 week after fetal surgery (26 + 3 weeks) (d–f), and approximately 6 weeks after fetal surgery (31 + 6 weeks) (g–i), in axial (a,d,g), coronal (b,e,h) and sagittal (c,f,i) planes.

A recently proposed automated SRR‐MRI algorithm^28^ was used to reconstruct an isotropic three‐dimensional (3D) fetal brain volume for each scan using at least three orthogonal T2‐weighted two‐dimensional (2D) MRI stacks in axial, coronal and sagittal orientations. The fetal brain was automatically localized and segmented in each 2D stack, followed by algorithmic motion correction and volumetric reconstruction steps. Iterative 3D reconstructions were estimated from motion‐corrected slices using outlier‐robust SRR methods to account for image artifacts and potential misalignments as part of the motion‐correction step. All images were reconstructed to an isotropic resolution of 0.8 mm. The obtained 3D reconstruction was aligned automatically to a spatiotemporal atlas in order to be presented in standard radiological anatomical orientation^37^. The quality of segmentations and reconstructions using this method had been previously assessed systematically by two expert pediatric radiologists, which showed that each step of this pipeline outperformed other state‐of‐the‐art methods^28^. We excluded patients owing to failed reconstructions at one or more timepoints (Figure 1). Reasons for failed reconstruction included excessive fetal motion artifacts in original stacks and/or between the stacks (*n* = 11) or brain imaging not performed in three orthogonal planes at the third timepoint using the recommended acquisition protocol (*n* = 5)^38^, ^39^. Figure 2 shows an example of the quality of the original data for an OSB case included in the final dataset.

### Automatic segmentation

Automatic segmentation of the unmyelinated white matter, ventricles and cerebellum was computed using deep‐learning‐based methods^40^, ^41^, ^42^. This provided good‐quality initial segmentation, which was subsequently corrected manually by a trained obstetrician (N.M.) and corrected as necessary thereafter by a consultant board‐certified pediatric neuroradiologist (M.A.) using ITK‐Snap™ (http://www.itksnap.org; version 3.20, 2014). After manual correction, meshes were generated as illustrated in Videoclip S1, ^43^. Manual refinement was necessary given the complexity and range of intracranial abnormalities observed in OSB, such as the direct apposition of the cortex to the skull, and the inability to differentiate between structures if compressed in a small posterior fossa owing to the Chiari‐II malformation. Unmyelinated white matter was defined as the brain parenchyma underneath the cortical plate and above the basal ganglia, which includes the intermediate zone, cortical subplate and ventricular zone. The ventricles were defined as the lateral, third and fourth ventricles, cerebral aqueduct, cavum septi pellucidi and cavum vergae. It was simpler to include the cavum septi pellucidi and vergae in ventricular parcellations, as we and others have observed several cases of OSB in which these structures were absent^2^, ^44^.

### Volumetric, shape and curvature‐based analysis

Using our generated meshes, a stratified analysis of cerebral and ventricular volume, followed by a global shape analysis (shape parameter) and a local surface‐based shape analysis (joint spectral matching), was carried out before and approximately 1 and 6 weeks after fetal surgery^2^. The longitudinal calculations between these timepoints were described as: (1) the immediate time period after fetal surgery (the difference between intrasubject meshes at 1 week after fetal surgery and before fetal surgery); (2) long‐term time period post surgery (the difference between intrasubject meshes at 6 weeks and 1 week after fetal surgery); and (3) the global time period (the difference between the long‐term and immediate time periods). Furthermore, we assessed these parameters according to lesion type (myeloschisis (MS) or myelomeningocele), postoperative persistence of hindbrain herniation and the presence of additional supratentorial anomalies, such as corpus callosum dysgenesis and heterotopia, identified on preoperative MRI and confirmed on postoperative MRI. Fetal MRI adds considerably to the evaluation of an anomalous corpus callosum^26^, showing that this structure can be visualized after 20 weeks' gestation. However, in order to reduce the subjectivity of corpus callosum assessment, all pre‐ and postoperative MRI assessments were performed and/or reported by highly experienced neuroradiologists (K.P.B., M.A.). These MRI acquisitions were performed and/or supervised by an expert MRI physicist (M.S.) in accordance with International Society of Ultrasound in Obstetrics and Gynecology fetal MRI guidelines, whereby images for the fetal brain were acquired in multiple orientations with at least one stack in axial, coronal and sagittal planes^39^.

The volume (mm^3^) was calculated as the sum of all voxels in each segmentation multiplied by the voxel dimension of the reconstructed volume. Surface area (mm^2^) was calculated as the sum of all triangular areas that composed each mesh. The global shape parameter (mm) was defined as volume/surface area.

As the fetal brain undergoes significant change in cortical gyrification with advancing gestation, we aimed to quantify this by using a surface‐based spectral‐matching technique^45^ to find the intrasubject longitudinal surface correspondences of the unmyelinated white matter in the immediate and long‐term time periods. Such correspondences serve as longitudinal measurements of change in cortical folding, provide information about the mechanical properties of the underlying tissue and may be particularly useful in inferring changes during a period of growth and development in which the fetal brain is vulnerable^45^. Joint spectral matching was therefore used to find the correspondence for the intrasubject cortical structures at these time periods^45^. A dual‐layered graph was produced in which the layers correspond to the cortical unmyelinated white‐matter surface in the immediate and long‐term time periods. The correspondence links from the initial intrasubject matching connected both layers and produced a set of shared eigenmode meshes with which we could compute parametric differences on highly folded cortical surfaces^45^, ^46^ (Figure S1).

**Figure 3:**
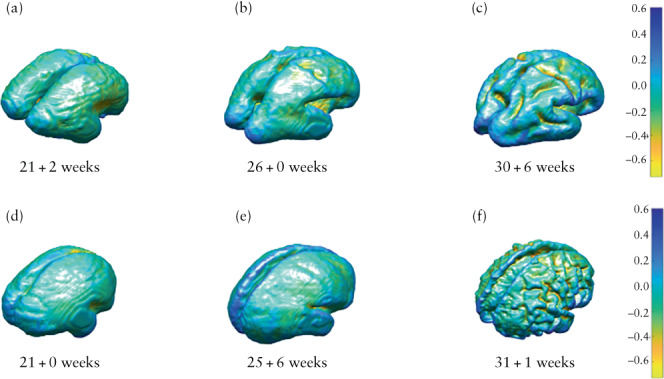
Mesh display (with accompanying color scale) of shape index for unmyelinated white matter in fetus with open spina bifida (d–f), compared with age‐matched control (a–c), assessed before fetal surgery (a,d), approximately 1 week after fetal surgery (b,e) and approximately 6 weeks after fetal surgery (c,f).

After mapping the two meshes in the immediate and long‐term time period using joint spectral matching, we computed the change in parameters at the vertex of each triangulated surface mesh to explore longitudinal gyrification^45^, ^47^. This was represented by mathematical markers of the curvature of the surface, namely curvedness (mm^–1^), which expresses the degree of deviation of cortical shapes from a flat plane (the extent to which a surface is curved), and the shape index (SI), which defines the convexity or concavity of the surface and can automatically locate gyral nodes and sulcal pits^48^, ^49^. Curvedness and SI are therefore mathematical references to the cortical morphology of the unmyelinated white‐matter surface and act as surrogate measures of cortical folding^27^. This concept is illustrated by the example SI meshes in Figure 3, in which positive values depicted in blue represent gyri (convex structures) and negative values in yellow represent sulci (concave structures). In order to ascertain quantitatively how different areas of the brain developed in the context of fetal surgery, we separated the cortical surface into frontal, temporal, occipital and parietal lobes, and further subdivided them into the right and left cerebral hemispheres. This was done by registering a normative spatiotemporal fetal brain MRI atlas template for the corresponding gestational age onto our unmyelinated white‐matter segmentations using non‐rigid registration^37^. Anatomical labels from the atlas were restructured manually to represent the four brain lobes in the two hemispheres^2^.

### Statistical analysis

Statistical analysis was performed using MATLAB (Mathworks Inc., Natick, MA, USA). When the Anderson–Darling test confirmed non‐normal distribution of data, the Kruskal–Wallis H‐test with correction for multiple comparisons was performed to compare differences in median values between groups. To account for any differences in gestational age, we compared the paired weekly rate of change in each parameter between the OSB and control fetuses at our three predefined timepoints; statistical significance was set at *P* < 0.05.

## RESULTS

### Demographics and cohort description

Twenty‐nine fetuses with OSB underwent MRI before (mean ± SD, 23 + 3 ± 1 + 1 (range, 21 + 0 to 25 + 6) weeks), approximately 1 week after (mean ± SD, 25 + 6 ± 0 + 5 (range, 24 + 1 to 26 + 2) weeks) and approximately 6 weeks after (mean ± SD, 31 + 6 ± 1 + 0 (range, 29 + 2 to 34 + 4) weeks) fetal surgery (Table S2). The results at each timepoint were compared with measurements from 12 corresponding age‐matched controls, whose mean ± SD gestational age was 23 + 1 ± 1 + 2 (range, 21 + 2 to 25 + 3) weeks at the first timepoint, 28 + 6 ± 1 + 3 (range, 26 + 0 to 30 + 1) weeks at the second timepoint and 32 + 6 ± 1 + 5 (range, 30 + 4 to 36 + 2) weeks at the third timepoint. There was no difference in gestational age between controls and OSB fetuses at baseline. However, there was a significant difference in gestational age between OSB fetuses and their corresponding controls at the MRI scans performed approximately 1 and 6 weeks after surgery (Table S2). The control fetuses had normal CNS findings on ultrasound, underwent MRI for non‐CNS indications (Appendix S1) and had normal CNS findings on MRI.

Twenty‐two (76%) patients had myelomeningocele and seven (24%) had MS. The lesion level started no higher than L1 and was no lower than S1 with hindbrain herniation present. The corpus callosum was abnormal in six (21%) cases, with a hypoplastic or dysplastic appearance. In 10 (34%) cases, there was partial agenesis of the corpus callosum (pACC) along its rostral–caudal axis, with the rostrum and/or splenium affected. This is in line with several postnatal studies in which the majority (57.1–95.9%) of children with OSB had varying degrees of corpus callosum anomaly^16^, ^50^, ^51^, ^52^. It is also in accordance with recent prenatal ultrasound and MRI studies that found that an abnormal corpus callosum was common (50.4–71.7%) in fetuses with isolated OSB that were referred for fetal surgery^5^, ^53^. In all cases in our cohort, the mesencephalic aqueduct was found to be patent before and after surgery. There was periventricular nodular heterotopia in seven (24%) cases, bilateral talipes in seven (24%) and absent or partial cavum septi pellucidi in eight (28%). There were four (14%) cases of mild (10–12 mm), eight (28%) of moderate (13–15 mm) and seventeen (59%) of severe (> 15 mm) ventriculomegaly measured at baseline MRI using the atrial diameter on the coronal ventricular plane^54^, ^55^, ^56^, ^57^.

### Volume and shape parameter

There was no difference in cerebellar volume growth per week and shape parameter change per week between OSB fetuses and controls during the three time periods. OSB cerebella appeared to follow a similar trajectory of shape‐parameter change to that of controls after surgery (Figure S2).

**Figure 4:**
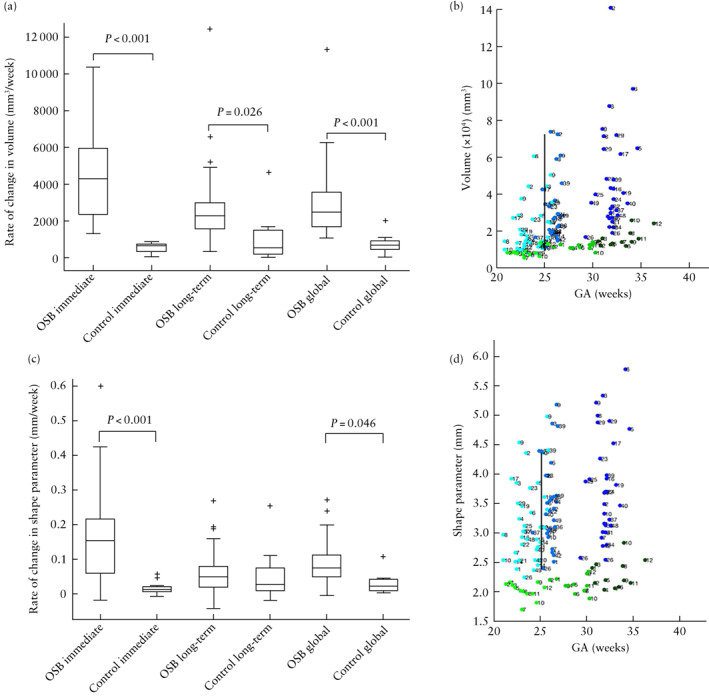
(a,c) Box‐and‐whiskers plots displaying rate of change in volume (a) and shape parameter (c) of ventricles in fetuses after open spina bifida (OSB) surgery compared with gestational age (GA)‐matched controls during immediate time period (difference between 1 week after fetal surgery and before fetal surgery), long‐term time period (difference between 6 weeks and 1 week after fetal surgery) and global time period (difference between long‐term and immediate time periods). Boxes with internal lines are median and interquartile range (IQR), whiskers are range and crosses are data points lying outside 1.5 × IQR. Only significant comparisons within time periods are indicated. (b,d) Scatterplots displaying volume (b) and shape parameter (d) of ventricles before (Timepoint 1 (T1)), 1 week after (Timepoint 2 (T2)) and 6 weeks after (Timepoint 3 (T3)) fetal surgery, in fetuses with OSB (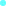, T1; 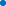, T2; 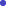, T3) and GA‐matched controls (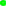, T1; 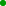, T2; 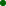, T3). Average GA at surgery indicated by black vertical line.

By contrast, the increase in ventricular volume per week, and thus the weekly rate of increase in intraventricular CSF, was significantly higher in OSB patients after surgery compared with controls in the immediate (median, 4326.67 (interquartile range (IQR), 2375.50–5965.90) mm^3^/week *vs* 681.02 (IQR, 359.60–753.30) mm^3^/week; *P* < 0.001), long‐term (median, 2315.18 (IQR, 1586.70–3006.00) mm^3^/week *vs* 580.05 (IQR, 211.40–1528.60) mm^3^/week; *P* = 0.026) and global (median, 2500.94 (IQR, 1689.70–3580.80) mm^3^/week *vs* 708.21 (IQR, 474.50–925.00) mm^3^/week; *P* < 0.001) time periods (Figure 4). Similarly, the change in ventricular shape parameter per week was significantly higher in OSB patients after surgery compared with controls in the immediate (median, 0.152 (IQR, 0.053–0.219) mm/week *vs* 0.012 (IQR, 0.006–0.022) mm/week; *P* < 0.001) and global (median, 0.075 (IQR, 0.047–0.112) mm/week *vs* 0.022 (IQR, 0.009–0.042) mm/week; *P* = 0.046) time periods (Figure 4).

**Figure 5:**
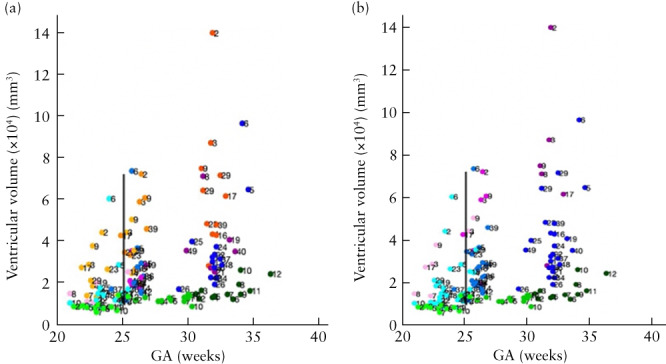
Scatterplots displaying ventricular volume before (Timepoint 1 (T1)), 1 week after (Timepoint 2 (T2)) and 6 weeks after (Timepoint 3 (T3)) fetal surgery, in fetuses with open spina bifida (OSB) and additional central nervous system (CNS) anomaly, OSB fetuses without additional CNS anomaly (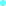, T1; 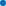, T2; 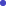, T3) and gestational age (GA)‐matched controls (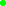, T1; 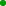, T2; 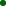, T3). Additional CNS anomalies were: (a) partial agenesis of corpus callosum (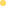, T1; 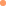, T2; 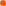, T3) or abnormal corpus callosum (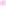, T1; 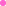, T2; 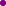, T3), and (b) persistent hindbrain herniation (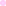, T1; 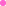, T2; 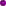, T3). Average GA at surgery indicated by black vertical line.

Ventricular volume growth per week after surgery in OSB patients with pACC was significantly higher than that in controls in the long‐term (median, 2759.98 (IQR, 2195.37–4999.88) mm^3^/week; *P* = 0.049) and global (median, 3177.91 (IQR, 2534.65–5341.50) mm^3^/week; *P* < 0.001) time periods, more so than OSB patients without pACC (Figure 5). Likewise, ventricular volume growth per week after surgery in OSB patients with persistent postoperative hindbrain herniation was significantly higher than that in controls in the immediate (median, 5421.10 (IQR, 3584.78–8188.76) mm^3^/week; *P* < 0.001), long‐term (median, 4260.84 (IQR, 2195.37–8058.02) mm^3^/week; *P* = 0.011) and global (median, 5017.11 (IQR, 2718.55–7548.02) mm^3^/week; *P* = 0.002) time periods, more so than OSB patients without persistent hindbrain herniation postoperatively (Figure 5).

**Figure 6:**
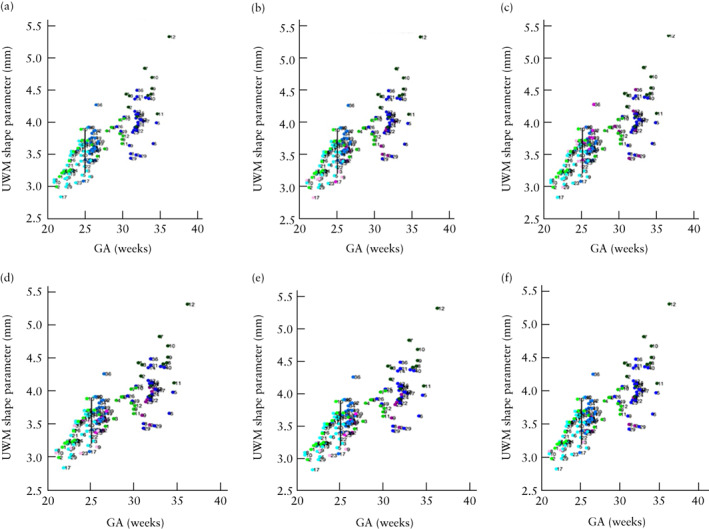
Scatterplots displaying unmyelinated white matter (UWM) shape parameter, before (Timepoint 1 (T1)), 1 week after (Timepoint 2 (T2)) and 6 weeks after (Timepoint 3 (T3)) fetal surgery, in fetuses with open spina bifida (OSB) and specific lesion type/additional central nervous system (CNS) anomaly (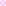, T1; 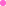, T2; 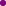, T3), OSB fetuses without additional CNS anomaly (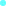, T1; 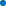, T2; 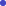, T3) and gestational age (GA)‐matched controls (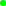, T1; 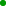, T2; 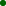, T3). Specific lesion type/additional CNS anomalies were: (b) persistent hindbrain herniation, (c) myeloschisis, (d) heterotopia, (e) heterotopia and corpus callosum anomaly and (f) persistent hindbrain herniation and corpus callosum anomaly. Average GA at surgery indicated by black vertical line.

Change in unmyelinated white‐matter shape parameter per week was lower in OSB fetuses compared with controls in the long‐term time period after surgery (median, 0.056 (IQR, 0.044–0.092) mm/week *vs* 0.159 (IQR, 0.100–0.247) mm/week; *P* = 0.002) (Figure 6). Furthermore, compared with controls, the change in unmyelinated white‐matter shape parameter per week was lower in the long‐term time period in OSB fetuses with persistent hindbrain herniation (median, 0.052 (IQR, 0.017–0.113) mm/week; *P* = 0.011), those with MS (median, 0.049 (IQR, 0.032–0.084) mm/week; *P* = 0.015), those with heterotopia (median, 0.053 (IQR, 0.019–0.073) mm/week; *P* = 0.022), those with heterotopia and corpus callosum abnormality (median, 0.056 (IQR, 0.041–0.087) mm/week; *P* = 0.017) and those with persistent hindbrain herniation and corpus callosum abnormality (median, 0.018 (IQR, 0.015–0.053) mm/week; *P* = 0.007). Additionally, OSB fetuses with persistent hindbrain herniation and corpus callosum abnormality had a lower change in shape parameter per week compared with controls in the global time period (median, 0.040 (IQR, 0.029–0.085) mm/week *vs* 0.100 (IQR, 0.085–0.140) mm/week; *P* = 0.047).

### Curvedness and shape index

**Figure 7:**
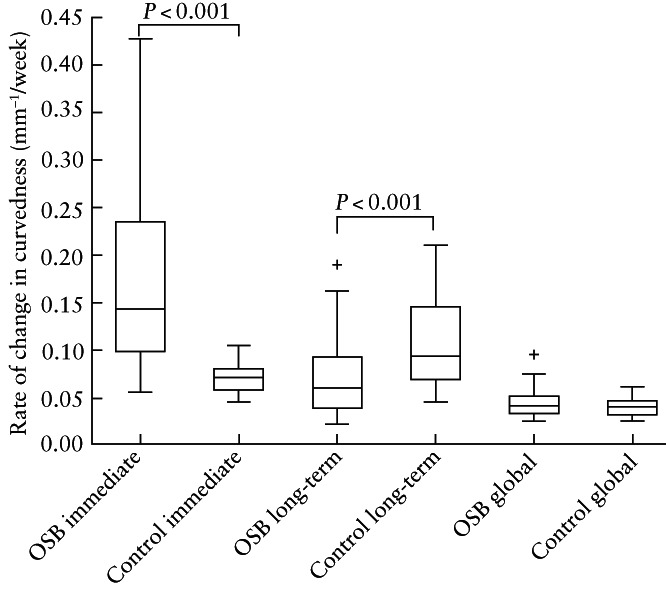
Box‐and‐whiskers plot displaying rate of change in curvedness of unmyelinated white matter in fetuses after open spina bifida (OSB) surgery compared with gestational age‐matched controls during immediate, long‐term and global time periods. Boxes with internal lines are median and interquartile range (IQR), whiskers are range and crosses are data points lying outside 1.5 × IQR. Only significant comparisons within time periods are indicated.

**Figure 8:**
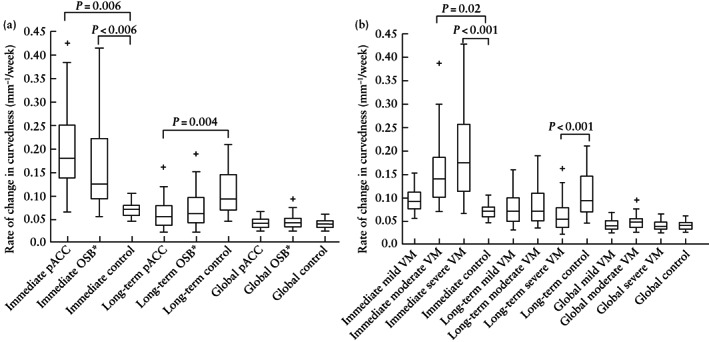
Box‐and‐whiskers plots displaying rate of change in curvedness of unmyelinated white matter after open spina bifida (OSB) surgery in fetuses with and those without partial agenesis of corpus callosum (pACC) (a) and fetuses with ventriculomegaly (VM) of varying severity (b), compared with gestational age‐matched controls during immediate time period (difference between 1 week after fetal surgery and before fetal surgery), long‐term time period (difference between 6 weeks and 1 week after fetal surgery) and global time period (difference between long‐term and immediate time periods). Boxes with internal lines are median and interquartile range (IQR), whiskers are range and crosses are data points lying outside 1.5 × IQR. Only significant comparisons with controls within time periods are indicated. *OSB fetuses without pACC.

The change in curvedness per week in OSB fetuses, compared with controls, was increased in the immediate time period (median, 0.144 (IQR, 0.099–0.236) mm^–1^/week *vs* 0.072 (IQR, 0.059–0.081) mm^–1^/week; *P* < 0.001) and decreased thereafter in the long‐term time period (median, 0.061 (IQR, 0.040–0.093) mm^–1^/week *vs* 0.094 (IQR, 0.070–0.146) mm^–1^/week; *P* < 0.001) (Figure 7). The change in curvedness per week in the immediate time period was increased, compared with controls, in OSB fetuses with pACC (median, 0.182 (IQR, 0.140–0.249) mm^–1^/week; *P* = 0.006) and OSB fetuses with severe ventriculomegaly (median, 0.175 (IQR, 0.115–0.257) mm^–1^/week; *P* < 0.001), more so than in OSB fetuses without these additional anomalies (Figure 8). Compared with controls, the change in curvedness per week in the long‐term time period was decreased in OSB fetuses with pACC (median, 0.057 (IQR, 0.037–0.078) mm^–1^/week; *P* = 0.004) and those with severe ventriculomegaly (median, 0.054 (IQR, 0.037–0.078) mm^–1^/week; *P* < 0.001).

**Figure 9:**
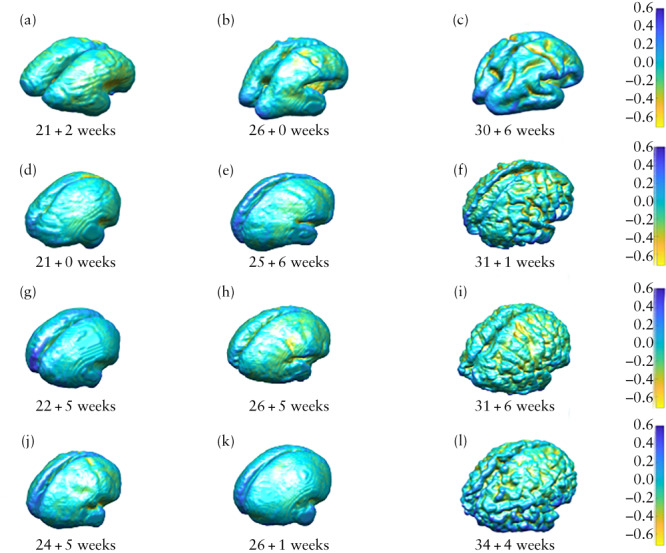
Mesh display (with accompanying color scale) of shape index for unmyelinated white matter in fetus with open spina bifida (OSB) (d–f), fetus with OSB and partial agenesis of corpus callosum (g–i), fetus with OSB and severe ventriculomegaly (j–l) and gestational age‐matched control (a–c), assessed before fetal surgery (a,d,g,j), approximately 1 week after fetal surgery (b,e,h,k) and approximately 6 weeks after fetal surgery (c,f,i,l).

Despite a notable reduction in the rate of change in curvedness 6 weeks after surgery compared with the immediate time period in OSB fetuses, OSB fetuses with pACC and those with severe ventriculomegaly, the SI mesh illustrations nonetheless show a resulting increase in the gyrification pattern compared with controls after fetal surgery (Figure 9). Furthermore, this finding was found to be maintained when unmyelinated white matter was separated into the frontal, parietal, temporal and occipital lobes and divided into the right and left hemispheres in OSB fetuses, those with pACC and those with severe ventriculomegaly (Tables S3–S5).

## DISCUSSION

### Principal findings

In this study, we applied advanced post‐processing MRI technology to analyze longitudinal cerebral development in the context of fetal OSB surgery. We found that, at 6 weeks after fetal surgery, the rate of ventricular growth, rate of change in ventricular and white‐matter shape parameters and rate of change in mathematical markers of gyrification differed significantly from those of control fetuses without OSB, and that these differences were particularly marked in those OSB cases with additional brain anomalies.

### Interpretation

We observed that ventriculomegaly does not reduce in the 6‐week period after fetal surgery. This is consistent with the results of other studies in which progressive ventricular widening was shown at 1 and 6 weeks after fetal surgery^56^, ^58^. The physiology of fetal CSF hydrodynamics in the prenatal setting has not been well investigated, but we postulate that there may be impaired CSF absorption following fetal surgery due to the lag in maturation of absorption pathways to accommodate the increased CSF load after OSB closure^2^, ^59^. Furthermore, we demonstrated that ventriculomegaly is higher 6 weeks after fetal surgery in OSB cases with pACC or persistent hindbrain herniation. This is in line with other studies that have shown an association between severe ventriculomegaly and corpus callosum anomalies, and that the persistence of hindbrain herniation after fetal surgery independently predicts the need for hydrocephalus treatment postnatally^5^, ^53^, ^58^, ^60^.

Furthermore, our study shows that change in unmyelinated white‐matter shape parameter per week was reduced 6 weeks after fetal surgery. This observation was maintained in OSB fetuses with MS, those with persistent hindbrain herniation after surgery and those with additional abnormalities, such as heterotopia, heterotopia with corpus callosum anomaly and persistent hindbrain herniation with corpus callosum anomaly. Reduction in the rate of change of the shape parameter could be due to the increase in surface area that occurs when the cortical surface folds compactly owing to cortical thinning associated with hydrocephalus, whereby atypical cortical gyri are formed by unfolding of the intrasulcal cortex onto the brain surface (Figure S3)^49^, ^59^. Hydrocephalus is particularly apparent in cases of OSB with persistent hindbrain herniation after fetal surgery, in which there is continual downward displacement of the cerebellum. Furthermore, CSF circulation is vital for neuronal migration, which can be disturbed by the mechanical forces from ventricular expansion^61^. This might explain the reduction in unmyelinated white‐matter shape parameter change per week seen in our OSB cohort with supratentorial anomalies^5^, ^10^, ^15^, ^62^.

Finally, we found an overall increase in gyrification in the 6‐week period after fetal surgery, particularly in OSB cases with pACC and those with severe ventriculomegaly, compared with control fetuses. This was despite a reduced curvedness per week in the long‐term time period compared with the increased rate in the immediate time period after fetal OSB surgery, which may be due to the effects of fetal closure. These cortical surface changes may be multifactorial. Atypical cortical organization may be related to the effects of hydrocephalus causing gradual destruction of white matter and reduced neuronal migration^9^, ^10^. Alternatively, or additionally, atypical cortical organization in OSB may be due to a primary maladaptive process that disrupts neuroembryogenesis and initiates a cascade of intracranial events, such as corpus callosum dysgenesis, heterotopia and polymicrogyria^5^, ^8^, ^9^, ^10^, ^11^, ^12^, ^13^, ^15^, ^16^, ^53^, ^60^, ^61^, ^63^, ^64^, ^65^. This would explain our observation that OSB fetuses with severe ventriculomegaly and those with pACC exhibited an increase in gyrification. These findings are in keeping with previous quantitative MRI studies that identified abnormal patterns of cortical thickening, thinning and gyrification in individuals who had undergone postnatal OSB closure compared with controls^8^, ^9^, ^10^. Regions that were thicker and more gyrified tended to be associated with poorer cognitive and motor function^8^, ^9^, ^10^. Improved understanding of the trajectories of brain development following prenatal OSB surgery and their associations when combined with additional supratentorial abnormalities may have functional impact on postnatal outcome, which could improve candidate selection in the future. It could also be an important tool for planning targeted treatment postnatally. Focused rehabilitation in early infancy is prudent, as it is a critical period of brain growth, maturation and intellectual development, during which intervention could optimize cognitive and motor function^14^, ^29^.

### Further research

Studies comparing our findings with those in fetuses with OSB that are eligible for fetal surgery but for which the parents opted for postnatal closure would provide a better understanding of the effects of fetal surgery on cortical development. White‐matter and cellular damage due to hydrocephalus depends on the duration and rate of ventricular enlargement^59^, ^62^. Fetal surgery may diminish this process, although further comparative studies are required to confirm this. However, our experience is that couples whose fetus is eligible for fetal surgery tend to proceed with this option, and only a small proportion choose postnatal closure. This makes it challenging to recruit sufficient numbers for a matched control group of OSB fetuses that have not undergone fetal surgery. Furthermore, postnatal follow‐up studies involving detailed motor, urological, neurocognitive and behavioral testing would clarify the clinical consequences of the white‐matter changes we observed.

### Strengths and limitations

A strength of our study is its application of state‐of‐the‐art MRI technology to explore longitudinal cerebral development in the context of fetal OSB surgery. Previous studies have assessed changes in brain morphology, such as gyrification, in children and adolescents who had postnatal OSB surgery^9^, ^10^, but we have adapted these techniques to evaluate morphological changes specifically in the fetal brain, in the context of fetal surgery. Furthermore, we analyzed developmental differences according to the presence of additional supratentorial anomalies and features of the lesion itself. We also studied fetal longitudinal intracranial development after OSB surgery, which captures the maturation of cortical gyrification that is dependent on gestational age and is best seen on MRI at 30–32 weeks^66^.

The main limitation of our study is that the control group consisted of fetuses with various non‐CNS anomalies. We also used three control groups comprising different fetuses to enable comparison with OSB cases at three gestational timepoints. This was owing to the challenging nature of obtaining a group of controls with longitudinal data. Although our data were acquired prospectively from multiple centers, another limitation of this study is the different MRI acquisition protocols used at the regional FMU referral centers compared to the FSCs, which share a common protocol. This may have affected the overall number of successful MRI reconstructions, as successful reconstruction depends on the quality of the original MRI data.

### Conclusions

In fetuses undergoing surgery for OSB, advanced MRI reconstruction techniques indicated longitudinal differences in volume and shape of the cerebellum, ventricles and unmyelinated white matter, as well as aberrant cortical folding, compared to control fetuses without OSB. These parameters differed according to the presence of supratentorial anomalies, type of lesion and persistence of hindbrain herniation after surgery. Our study offers a new perspective on the implications of fetal OSB surgery, in that there are changes in brain development, in terms of white‐matter maturation and gyrification, that accompany the improved CSF dynamics following fetal repair.

## COLLABORATORS

### GIFT‐Surg Imaging Working Group

David Atkinson, Centre for Medical Imaging, UniversityCollege London, London, UK

Foteini Emmanouella Bredaki, Women's Health Division,University College London Hospital, London, UK

Luc De Catte, Department of Obstetrics and Gynaecology,University Hospitals Katholieke Universiteit (KU)Leuven, Leuven, Belgium

Phillippe De Vloo, Department of Neurosurgery, University Hospitals Katholieke Universiteit (KU) Leuven,Leuven, Belgium

Philippe Demaerel, Department of Radiology, UniversityHospitals Katholieke Universiteit (KU) Leuven, Leuven, Belgium

Roland Devlieger, Department of Obstetrics and Gynaecology, University Hospitals Katholieke Universiteit(KU) Leuven, Leuven, Belgium

Trevor Gaunt, Radiology Department, Great OrmondStreet Hospital for Children, London, UK

Giles S. Kendall, Women's Health Division, UniversityCollege London Hospital, London, UK; ElizabethGarrett Anderson Institute for Women's Health,University College London, London, UK

Sebastien Ourselin, School of Biomedical Engineering andImaging Sciences (BMEIS), King's College London,London, UK; Medical Physics and Biomedical Engineering, University College London, London, UK

Kelly Pegoretti Baruteau, Radiology Department, University College London Hospital, London, UK

Adalina Sacco, Women's Health Division, UniversityCollege London Hospital, London, UK; Elizabeth Garrett Anderson Institute for Women's Health, UniversityCollege London, London, UK

Magdalena Sokolska, Department of Medical Physicsand Biomedical Engineering, University College London Hospital, London, UK

Dominic Thompson, Paediatric Neurosurgery Department, Great Ormond Street Hospital for Children,London, UK

Tom Vercauteren, School of Biomedical Engineering andImaging Sciences (BMEIS), King's College London,London, UK; Medical Physics and Biomedical Engineering, University College London, London, UK

## Supporting information


**Table S1** Parametric comparisons for sequences used in super‐resolution reconstruction of magnetic resonance images of fetal brain in fetal surgery centers and regional fetal medicine unit referral centers
**Table S2** Gestational age of cases and controls at time of magnetic resonance imaging
**Table S3** Change in curvedness per week of whole brain and lobes in both hemispheres in fetuses with open spina bifida and gestational age‐matched controls
**Table S4** Change in curvedness per week of whole brain and lobes in both hemispheres in fetuses with open spina bifida and partial agenesis of corpus callosum
**Table S5** Change in curvedness per week of whole brain and lobes in both hemispheres in fetuses with open spina bifida and severe ventriculomegaly
**Figure S1** First five spectral modes of unmyelinated white matter in fetus at three timepoints: before fetal surgery (top row), approximately 1 week after fetal surgery (middle row) and approximately 6 weeks after fetal surgery (bottom row). Colors represent spatial values of first five eigenmodes. Each eigenmode typically encodes the dominant spatial frequencies across the cohort, moving from low spatial frequencies (mainly primary sulci) to high spatial frequencies (tertiary sulci and finer). Eigenmodes are normalized so have a magnitude of 1; most negative values are blue and most positive values are red. Although eigenmode meshes are significantly different in three‐dimensional space, with respect to different levels of folding and variation in shape, surface area and volume, they have similar representations in the spectral domain. This makes the two surfaces comparable so it is easier to map a good qualitative correspondence between them, allowing measurement of longitudinal changes that take place in this region.
**Figure S2** Scatter plots displaying volume (a), surface area (b) and shape parameter (c) of cerebellum in fetuses with open spina bifida (OSB) before, at 1 week after and at 6 weeks after surgery, compared to age‐matched controls.
**Figure S3** Mesh display (with accompanying color scale) of shape index for unmyelinated white matter in control fetus (first row) compared to fetus with myeloschisis (MS) (second row), fetus with open spina bifida (OSB) and persistent postoperative hindbrain herniation (HH) (third row), fetus with OSB and heterotopia (HT) (fourth row), fetus with OSB, HT and corpus callosum (CC) abnormality (fifth row) and fetus with OSB, persistent postoperative HH and CC abnormality (sixth row), assessed before fetal surgery (a), approximately 1 week after fetal surgery (b) and approximately 6 weeks after fetal surgery (c).
**Appendix S1** Indications for fetal magnetic resonance imaging in controls

**Videoclip S1** Animation showing four meshes generated from magnetic resonance images of same fetus with open spinal bifida, in order of increasing gestational age, in context of fetal surgery (timing indicated by brown dashed line). Purple structure depicts unmyelinated white matter, yellow depicts ventricles and green depicts cerebellum. Surfaces of first two unmyelinated white‐matter meshes show primary gyrification and those of last two meshes display secondary gyrification.

## Data Availability

Data available on request from the authors.
