# Supplementary material for: Assessment of longitudinal brain development using super‐resolution magnetic resonance imaging following fetal surgery for open spina bifida
Source: Ultrasound Obstet Gynecol. 2023 Nov 1;62(5):707–20. doi: 10.1002/uog.26244 (PMC10947002; doi:10.1002/uog.26244)

| Sequence | Centre | Slice Thickness (mm)  Mean ± Standard Deviation, (Range) | Spacing Between Slices (mm) Mean ± Standard Deviation, (Range) | Echo Time (ms) Mean ± Standard Deviation, (Range) | Repetition Time (ms) Mean ± Standard Deviation, (Range) | Pixel Bandwidth (Hz)  Mean ± Standard Deviation, (Range) | Pixel Spacing (mm)  Mean ± Standard Deviation, (Range) | Flip Angle°  Mean ± Standard Deviation, (Range) | SAR  Mean ± Standard Deviation, (Range) |
| --- | --- | --- | --- | --- | --- | --- | --- | --- | --- |
| T2 HASTE Brain | Fetal Surgery Centre | 3.42 ± 0.18, (3-3.5) | 3.56 ± 0.24, (3-3.68) | 135.73 ± 4.91, (2.38-139) | 1094.42 ± 67.33, (4.75-1330) | 262.62 ± 106.44, (195-475) | (0.92, 0.92) ± 0.17, (0.59-1.25) | 133.66 ± 17.04, (70-150) | 1.57 ± 0.23, (1.1-2) |
|  | Regional Fetal Medicine Unit Referral Centre | 3.96 ± 0.37, (3-5) | 4.15 ± 0.43, (3.3 -5) | 140.78 ± 26.6, (63-167) | 2281.32 ± 2920.72, (1000-13679.3) | 322.11 ± 91.15, (195-501) | (1.05, 1.05) ± 0.19, (0.56-1.25) | 132 ± 19.85, (90-150) | 1.61 ± 0.17, (1.38-2) |
| SSFSE Brain | Regional Fetal Medicine Unit Referral Centre | 3.62 ± 0.48, (3-4) | 3.86 ± 0.68, (3-4.4) | 136.84 ± 26.39, (92.35-240) | 15136.66 ± 11477.64, (1000-29488.44) | 346.01 ± 111.87, (122.07-488.28) | (1.15, 1.15) ± 0.18, (0.74-1.25) | 90 | 1.42 ± 0.64, (0.31-1.91) |
| TSE Brain | Regional Fetal Medicine Unit Referral Centre | 3.88 ± 0.34, (3-4) | 4.26 ± 0.38, (3.3-4.4) | 140 | 23079.71 ± 8407.63, (10063.03-40105.92) | 307.38 ± 41.97, (202-334) | (1.19, 1.19) ± 0.15, (0.8-1.25) | 90 | 1.91 |

**Table S1**  Parametric comparisons for sequences used in super-resolution reconstruction of magnetic resonance images of fetal brain in fetal surgery centers and regional fetal medicine unit referral centers

**Figure S1**  First five spectral modes of unmyelinated white matter in fetus at three timepoints: before fetal surgery (top row), approximately 1 week after fetal surgery (middle row) and approximately 6 weeks after fetal surgery (bottom row). Colors represent spatial values of first five eigenmodes. Each eigenmode typically encodes the dominant spatial frequencies across the cohort, moving from low spatial frequencies (mainly primary sulci) to high spatial frequencies (tertiary sulci and finer). Eigenmodes are normalized so have a magnitude of 1; most negative values are blue and most positive values are red. Although eigenmode meshes are significantly different in three-dimensional space, with respect to different levels of folding and variation in shape, surface area and volume, they have similar representations in the spectral domain. This makes the two surfaces comparable so it is easier to map a good qualitative correspondence between them, allowing measurement of longitudinal changes that take place in this region.


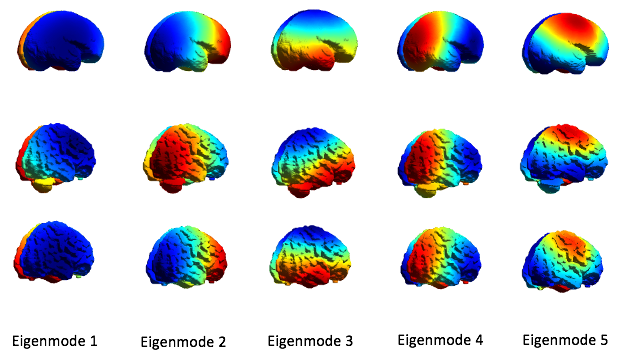


**Table S2** Gestational age of cases and controls at time of magnetic resonance imaging

| MRI Timing | OSB (n=29), mean and range ± SD GA (weeks + days) | Controls (n=12), mean and range ± SD GA (weeks + days) | AD-Crit* |
| --- | --- | --- | --- |
| Before Surgery | 23+3 (21+0 - 25+6) ± 1+1 | 23+1 (21+2 - 25+3) ± 1+2^†^ | 0.559 |
| ~1 Week After Surgery | 25+6 (23+1 - 26+2) ± 0+5 | 28+6 (26+0 - 30+1) ± 1+3^‡^ | 15.547 |
| ~6 Weeks After Surgery | 31+6 (29+2 - 34+4) ± 1+0 | 32+6 (30+4 -36+2) ± 1+5^§^ | 3.617 |

OSB, Open Spina Bifida; SD, standard deviation; GA, gestational age

*Anderson darling criteria (AD Crit) =0.738 The null hypothesis that x and y come from the same continuous distribution is rejected if AD ≥ AD Crit where AD Crit is the critical value found in the table of critical values. So, one-week, and six-week post-operative MRI’s between OSB and Controls do not come from the same continuous distribution hence the requirement to use rate of parameter change/week.

**Appendix S1** Indications for fetal magnetic resonance imaging in controls

Before Surgery age-matched control case indications for fetal MRI: kidney cyst, trachea-oesophageal fistula, arthrogryposis and micrognathia, vascular liver malformation, placenta praevia, epidermal inclusion cyst, maternal cervical teratoma, urogenital malformation, large choledochus cyst, fetal scoliosis, hyperechogenic lung, gastroschisis

One-week post-surgery age-matched control case indications for fetal MRI: enlarged thyroid gland, pelvic ureteric junction obstruction, laryngo-oesophageal fistula, sacrococcygeal teratoma, lymphangioma, abnormally invasive placenta, Liver mass, hyperflexion of both hands, talipes, limb reduction defect

Six weeks post-surgery age-matched control case indications for fetal MRI: polyhydramnios, low urinary tract obstruction, lymphangioma, placenta accreta spectrum disorder, Rokitansky syndrome, intestinal cysts.

**Figure S2**  Scatter plots displaying volume (a), surface area (b) and shape parameter (c) of cerebellum in fetuses with open spina bifida (OSB) before, at 1 week after and at 6 weeks after surgery, compared to age-matched controls.


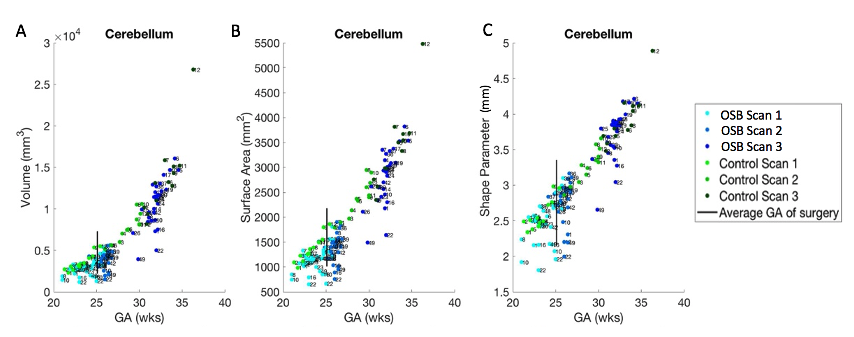


**Table S3** Change in curvedness per week of whole brain and lobes in both hemispheres in fetuses with open spina bifida and gestational age-matched controls

|  | **Immediate Time Period** | | | **Long Term Time Period** | | |
| --- | --- | --- | --- | --- | --- | --- |
|  | **OSB**  **Median, IQR range** | **Control**  **Median, IQR range** | ***P-Value*** | **OSB**  **Median, IQR range** | **Control**  **Median, IQR range** | ***P-Value*** |
| **Whole Brain** | 0.144 (IQR: 0.101-0.233) | 0.072 (IQR: 0.059-0.08) | ****<0.001*** | 0.061 (IQR: 0.040-0.093) | 0.094 (IQR: 0.072-0.145) | ****<0.001*** |
| **Left Frontal** | 0.151 (IQR: 0.1-0.232) | 0.073 (IQR: 0.063-0.086) | ****<0.001*** | 0.065 (IQR: 0.054-0.079) | 0.099 (IQR: 0.082-0.118) | ****<0.001*** |
| **Right Frontal** | 0.15 (IQR: 0.103-0.241) | 0.074 (IQR: 0.065-0.083) | ****<0.001*** | 0.066 (IQR: 0.055-0.07) | 0.099 (IQR: 0.084-0.116) | ****<0.001*** |
| **Left Parietal** | 0.158 (IQR: 0.116-0.253) | 0.076 (IQR: 0.065-0.082) | ****<0.001*** | 0.068 (IQR: 0.057-0.082) | 0.1 (IQR: 0.085-0.119) | ****<0.001*** |
| **Right Parietal** | 0.153 (IQR: 0.109-0.249) | 0.072 (IQR: 0.063-0.089) | ****<0.001*** | 0.068 (IQR: 0.057-0.082) | 0.102 (IQR: 0.087-0.123) | ****<0.001*** |
| **Left Temporal** | 0.159 (IQR: 0.106-0.261) | 0.075 (IQR: 0.065-0.087) | ****<0.001*** | 0.069 (IQR: 0.057-0.082) | 0.1 (IQR: 0.084-0.119) | ****0.001*** |
| **Right Temporal** | 0.163 (IQR: 0.111-0.268) | 0.075 (IQR: 0.064-0.086) | ****<0.001*** | 0.068 (IQR: 0.057-0.085) | 0.103 (IQR: 0.082-0.12) | ****0.002*** |
| **Left Occipital** | 0.146 (IQR: 0.102-0.22) | 0.072 (IQR: 0.06-0.079) | ****<0.001*** | 0.06 (IQR: 0.05-0.074) | 0.095 (IQR: 0.077-0.109) | ****<0.001*** |
| **Right Occipital** | 0.143 (IQR: 0.104-0.235) | 0.071 (IQR: 0.062-0.083) | ****<0.001*** | 0.061 (IQR: 0.051-0.076) | 0.099 (IQR: 0.08-0.113) | ****<0.001*** |

OSB, Open Spina Bifida; IQR, inter-quartile range; *significant *p*-value

Rate of curvedness mm ^-1^/week for unmyelinated white matter for the whole brain, and all lobes in the right and left hemispheres. Significant results are seen for the whole brain across the immediate and long-term time periods.

**Table S4**  Change in curvedness per week of whole brain and lobes in both hemispheres in fetuses with open spina bifida and partial agenesis of corpus callosum

|  | **Immediate Time Period** | | | | | **Long Term Time Period** | | | | |
| --- | --- | --- | --- | --- | --- | --- | --- | --- | --- | --- |
|  | **Control** | **OSB** | | **pACC** | | **Control** | **OSB** | | **pACC** | |
| **Location of Brain** | **Median, IQR** | **Median, IQR** | **P-Value** | **Median, IQR** | **P-Value** | **Median, IQR** | **Median, IQR** | **P-Value** | **Median, IQR** | **P-Value** |
| **Left Frontal** | 0.073 (IQR: 0.063-0.086) | 0.129 (IQR: 0.096-0.227) | ****0.003*** | 0.2 (IQR: 0.147-0.266) | ****<0.001*** | 0.097 (IQR: 0.082-0.118) | X | X | 0.059 (IQR: 0.052-0.071) | ****0.001*** |
| **Right Frontal** | 0.074 (IQR: 0.065-0.083) | 0.124 (IQR: 0.099-0.228) | ****0.004*** | 0.185 (IQR: 0.146-0.272) | ****<0.001*** | 0.099 (IQR: 0.084-0.116) | 0.069 (IQR: 0.056-0.082) | ****0.042*** | 0.06 (IQR: 0.051-0.071) | ****0.001*** |
| **Left Parietal** | 0.076 (IQR: 0.065-0.082) | 0.143 (IQR: 0.102-0.239) | ***<0.001*** | 0.205 (IQR: 0.157-0.283) | ****<0.001*** | 0.1 (IQR: 0.085-0.119) | X | X | 0.064 (IQR: 0.05-0.077) | ****0.002*** |
| **Right Parietal** | 0.074 (IQR: 0.063-0.086) | 0.136 (IQR: 0.104-0.226) | ****0.001*** | 0.205 (IQR: 0.154-0.276) | ****<0.001*** | 0.102 (IQR: 0.087-0.123) | X | X | 0.061 (IQR: 0.055-0.078) | ****0.002*** |
| **Left Temporal** | 0.075 (IQR: 0.065-0.087) | 0.133 (IQR: 0.102-0.235) | ****0.002*** | 0.211 (IQR: 0.148-0.288) | ****<0.001*** | 0.101 (IQR: 0.084-0.119) | X | X | 0.0617 (IQR: 0.054-0.088) | ****0.004*** |
| **Right Temporal** | 0.075 (IQR: 0.064-0.086) | 0.137 (IQR: 0.103-0.253) | ****<0.001*** | 0.204 (IQR: 0.154-0.275) | ****<0.001*** | 0.103 (IQR: 0.082-0.12) | X | X | 0.062 (IQR: 0.051-0.077) | ****0.004*** |
| **Left Occipital** | 0.072 (IQR: 0.06-0.079) | 0.128 (IQR: 0.1-0.213) | ****0.002*** | 0.181 (IQR: 0.145-0.25) | ****<0.001*** | 0.1 (IQR: 0.077-0.109) | 0.062 (IQR: 0.053-0.077) | ****0.046*** | 0.056 (IQR: 0.053-0.046-0.068) | ****0.001*** |
| **Right Occipital** | 0.071 (IQR: 0.062-0.083) | 0.124 (IQR: 0.1-0.224) | ****<0.001*** | 0.185 (IQR: 0.142-0.248) | ****0.003*** | 0.1 (0.08-0.113) | 0.064 (IQR: 0.053-0.078) | ****0.036*** | 0.056 (IQR: 0.045-0.069) | ****0.001*** |

OSB, Open Spina Bifida; pACC, partial agenesis of the corpus callosum; IQR, inter-quartile range; *significant *p*-value

Rate of curvedness mm ^-1^/week for unmyelinated white matter for all lobes in the right and left hemispheres for OSB with pACC and those without. For pACC significant results are seen for the whole brain across the immediate and long-term time periods.

**Table S5**  Change in curvedness per week of whole brain and lobes in both hemispheres in fetuses with open spina bifida and severe ventriculomegaly

|  | **Immediate Time Period** | | | **Long Term Time Period** | | |
| --- | --- | --- | --- | --- | --- | --- |
|  | **Control** | **Severe Ventriculomegaly** | | **Control** | **Severe Ventriculomegaly** | |
| **Location of Brain** | **Median, IQR** | **Median, IQR** | **P-Value** | **Median, IQR** | **Median, IQR** | **P-Value** |
| **Left Frontal** | 0.074 (IQR: 0.063-0.086) | 0.218 (IQR: 0.119-0.303) | ****<0.001*** | 0.099 (IQR: 0.082-0.118) | 0.061 (IQR: 0.049-0.071) | ****0.001*** |
| **Right Frontal** | 0.075 (IQR: 0.065-0.083) | 0.215  (IQR: 0.121-0.291) | ****<0.001*** | 0.1 (IQR: 0.084-0.116) | 0.061 (IQR: 0.05-0.072) | ****0.001*** |
| **Left Parietal** | 0.075 (IQR: 0.065-0.082) | 0.23 (IQR: 0.132-0.309) | ****<0.001*** | 0.102 (IQR: 0.085-0.119) | 0.064 (IQR: 0.05-0.075) | ****<0.001*** |
| **Right Parietal** | 0.074 (IQR: 0.063-0.086) | 0.228 (IQR: 0.128-0.319) | ****<0.001*** | 0.104 (IQR: 0.087-0.123) | 0.064 (IQR: 0.052-0.077) | ****<0.001*** |
| **Left Temporal** | 0.077 (IQR: 0.065-0.087) | 0.224 (IQR: 0.129-0.306) | ****<0.001*** | 0.101 (IQR: 0.084-0.119) | 0.0632 (IQR: 0.049-0.076) | ****<0.001*** |
| **Right Temporal** | 0.076 (IQR: 0.064-0.086) | 0.224 (IQR: 0.128-0.295) | ****<0.001*** | 0.103 (IQR: 0.082-0.12) | 0.064 (IQR: 0.051-0.076) | ****<0.001*** |
| **Left Occipital** | 0.072 (IQR: 0.06-0.079) | 0.203 (IQR: 0.119-0.262) | ****<0.001*** | 0.095 (IQR: 0.077-0.109) | 0.057 (IQR: 0.045-0.068) | ****<0.001*** |
| **Right Occipital** | 0.073 (IQR: 0.062-0.083) | 0.206 (IQR: 0.121-0.268) | ****<0.001*** | 0.098 (0.08-0.113) | 0.058 (IQR: 0.044-0.068) | ****<0.001*** |

IQR, inter-quartile range; *significant *p*-value

Rate of curvedness mm ^-1^/week for unmyelinated white matter for all lobes in the right and left hemispheres for OSB with severe ventriculomegaly. Significant results are seen for the whole brain across the immediate and long-term time periods.

**Figure S3**  Mesh display (with accompanying color scale) of shape index for unmyelinated white matter in control fetus (first row) compared to fetus with myeloschisis (MS) (second row), fetus with open spina bifida (OSB) and persistent postoperative hindbrain herniation (HH) (third row), fetus with OSB and heterotopia (HT) (fourth row), fetus with OSB, HT and corpus callosum (CC) abnormality (fifth row) and fetus with OSB, persistent postoperative HH and CC abnormality (sixth row), assessed before fetal surgery (a), approximately 1 week after fetal surgery (b) and approximately 6 weeks after fetal surgery (c).


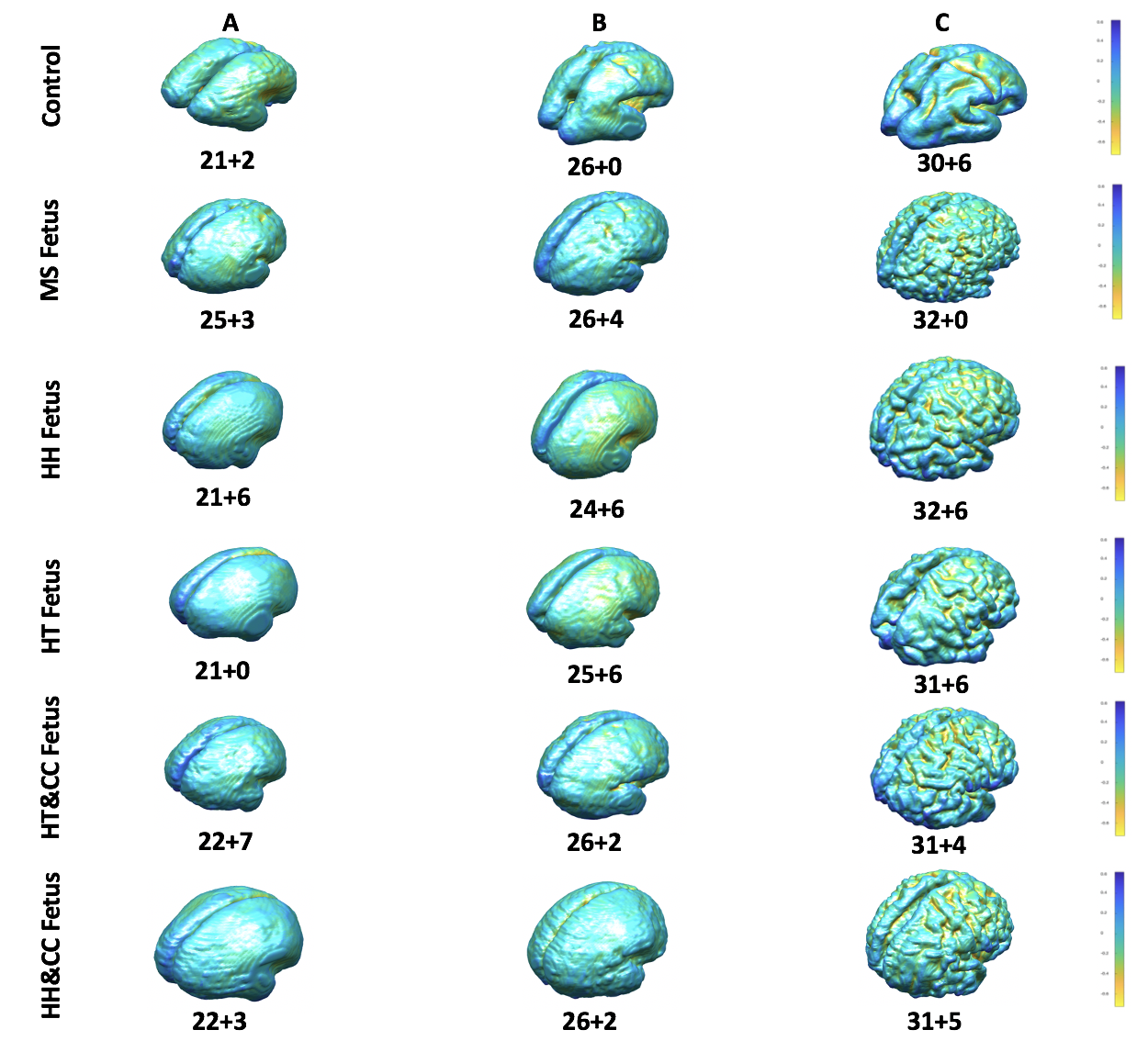

Supplement: Supplementary file 1 — Table S1 Parametric comparisons for sequences used in super‐resolution reconstruction of magnetic resonance images of fetal brain in fetal surgery centers and regional fetal medicine unit referral centers Table S2 Gestational age of cases and controls at time of magnetic resonance imaging Table S3 Change in curvedness per week of whole brain and lobes in both hemispheres in fetuses with open spina bifida and gestational age‐matched controls Table S4 Change in curvedness per week of whole brain and lobes in both hemispheres in fetuses with open spina bifida and partial agenesis of corpus callosum Table S5 Change in curvedness per week of whole brain and lobes in both hemispheres in fetuses with open spina bifida and severe ventriculomegaly Figure S1 First five spectral modes of unmyelinated white matter in fetus at three timepoints: before fetal surgery (top row), approximately 1 week after fetal surgery (middle row) and approximately 6 weeks after fetal surgery (bottom row). Colors represent spatial values of first five eigenmodes. Each eigenmode typically encodes the dominant spatial frequencies across the cohort, moving from low spatial frequencies (mainly primary sulci) to high spatial frequencies (tertiary sulci and finer). Eigenmodes are normalized so have a magnitude of 1; most negative values are blue and most positive values are red. Although eigenmode meshes are significantly different in three‐dimensional space, with respect to different levels of folding and variation in shape, surface area and volume, they have similar representations in the spectral domain. This makes the two surfaces comparable so it is easier to map a good qualitative correspondence between them, allowing measurement of longitudinal changes that take place in this region. Figure S2 Scatter plots displaying volume (a), surface area (b) and shape parameter (c) of cerebellum in fetuses with open spina bifida (OSB) before, at 1 week after and at 6 weeks after surgery, compared to age‐matc [file UOG-62-707-s001.docx]
